# Supplementary material for: No evidence of reduced capacity during highly demanding cognitive tasks in healthy older adults at electroencephalographic risk of cognitive impairment
Source: PLoS One. 2025 Apr 30;20(4):e0320722. doi: 10.1371/journal.pone.0320722 (PMC12043134; doi:10.1371/journal.pone.0320722)
Supplement: S1 Table — (DOCX) [file pone.0320722.s001.docx]

**S1 Table. Risk *vs*. Control group differences on the WAIS-IV**

|  |  | | **Risk** | | **Control** | **t-value** | | ***p*-FDR** | |  |
| --- | --- | --- | --- | --- | --- | --- | --- | --- | --- | --- |
|  |  | | Mean (SD) | | Mean (SD) |  | |  | |  |
| **Block design** | | 11.83(2.67) | | 10.29(2.23) | | | -2.29 | | 0.65 | |
| **Similarities** | | 12.75(2.18) | | 13.29(2.50) | | | 0.90 | | 0.96 | |
| **Digit span** | | 11.47(2.17) | | 11.25(1.91) | | | -0.40 | | 0.96 | |
| **DIG-F** | | 11.02(2.85) | | 10.51(2.22) | | | -0.88 | | 0.96 | |
| **DIG-B** | | 11.67(1.97) | | 10.85(1.56) | | | -1.79 | | 0.76 | |
| **Matrix reasoning** | | 12.83(1.97) | | 12(1.79) | | | -1.67 | | 0.87 | |
| **Vocabulary** | | 14.11(1.46) | | 13.55(1.39) | | | -1.47 | | 0.87 | |
| **Arithmetic** | | 11.75(2.54) | | 11.59(2.51) | | | -0.24 | | 0.97 | |
| **Symbol search** | | 11.86(1.64) | | 11.66(1.38) | | | -0.49 | | 0.96 | |
| **Visual puzzles** | | 11.36(2.17) | | 11(2.33) | | | -0.62 | | 0.96 | |
| **Information** | | 13.52(1.84) | | 13.14(1.95) | | | -0.77 | | 0.96 | |
| **Coding** | | 12.13(1.45) | | 12.14(1.35) | | | 0.04 | | 1.00 | |
| **VCI** | | 122.63(10.85) | | 121.77(11.10) | | | -0.31 | | 0.96 | |
| **PRI** | | 111.83(11.49) | | 106.03(11.10) | | | -1.93 | | 0.65 | |
| **WMI** | | 108.25(10.85) | | 107.18(10.05) | | | -0.39 | | 0.96 | |
| **PSI** | | 110.30(7.93) | | 109.70(7.25) | | | -0.28 | | 0.96 | |
| **FSIQ** | | 116.11(10.41) | | 113.(9.55) | | | -1.15 | | 0.25 | |

DIG-F = digit span recall forward; DIG-B: digit span recall backward; VCI: verbal comprehension index; PRI: perceptual reasoning index; WMI: working memory index; PSI: processing speed index; FSIQ: full scale IQ.
