# Supplementary material for: No evidence of reduced capacity during highly demanding cognitive tasks in healthy older adults at electroencephalographic risk of cognitive impairment
Source: PLoS One. 2025 Apr 30;20(4):e0320722. doi: 10.1371/journal.pone.0320722 (PMC12043134; doi:10.1371/journal.pone.0320722)
Supplement: S5 Table — (DOCX) [file pone.0320722.s005.docx]

**S5 Table. High risk *vs*. Low risk *vs*. Control group differences on the sum of zAP(theta) squared (maintaining the sign)**

| **Cognitive test** |  | **F-value** | ***p* value** |
| --- | --- | --- | --- |
|  |  | df(2,60) |  |
| **RCF** |  |  |  |
| **Copy** |  | 3.85 | 0.65 |
| **Retrieval** |  | 1.48 | 0.89 |
| **Logical memory** |  |  |  |
| **Encoding** |  | 0.49 | 0.96 |
| **Retrieval** |  | 0.09 | 0.97 |
| **PA** |  |  |  |
| **Encoding** |  | 0.78 | 0.96 |
| **Retrieval** |  | 0.44 | 0.96 |
| **Semantic fluency** |  | 0.61 | 0.96 |
| **Phonologic fluency** |  | 0.90 | 0.96 |
| **Maze planning** |  | 0.20 | 0.97 |
| **Hanoi 3** |  | 2.20 | 0.87 |
| **Hanoi 4** |  | 1.94 | 0.87 |
| **TMT-A** |  | 0.38 | 0.96 |
| **TMT-B** |  | 0.94 | 0.96 |
| **Stroop interference** |  | 0.79 | 0.96 |
| **STM Single 2** |  | 0.98 | 0.96 |
| **STM Single 3** |  | 0.67 | 0.96 |
| **STM Binding 2** |  | 0.56 | 0.96 |
| **STM Binding 3** |  | 1.40 | 0.89 |
| **Free recall A** |  | 0.45 | 0.96 |
| **Cued recall A** |  | 0.82 | 0.96 |
| **Cued recall A2 (maximum storage)** |  | 1.98 | 0.87 |
| **Free recall B** |  | 0.005 | 1.00 |
| **Cued recall B(PSI)** |  | 0.32 | 0.96 |
| **Cued recall B2(r-PSI)** |  | 1.29 | 0.92 |
| **Free recall A3** |  | 0.41 | 0.96 |
| **Cued recall A3(RSI)** |  | 0.11 | 0.97 |
| **Delayed recall** |  | 0.14 | 0.97 |
| **% of intrusion errors on cued recall B** |  | 0.01 | 1.00 |
| **% of intrusion errors on cued recall B2** |  | 1.90 | 0.87 |
| **Total intrusions** |  | 0.11 | 0.97 |
| **Block design** |  | 4.57 | 0.65 |
| **Similarities** |  | 0.73 | 0.96 |
| **Digit span** |  | 0.11 | 0.97 |
| **DIG-F** |  | 0.39 | 0.96 |
| **DIG-B** |  | 1.74 | 0.87 |
| **Matrix reasoning** |  | 1.51 | 0.89 |
| **Vocabulary** |  | 3.52 | 0.65 |
| **Arithmetic** |  | 0.04 | 0.98 |
| **Symbol search** |  | 0.26 | 0.96 |
| **Visual puzzles** |  | 0.52 | 0.96 |
| **Information** |  | 0.77 | 0.96 |
| **Coding** |  | 0.34 | 0.96 |
| **VCI** |  | 0.97 | 0.96 |
| **PRI** |  | 2.80 | 0.76 |
| **WMI** |  | 0.08 | 0.97 |
| **PSI** |  | 0.04 | 0.98 |
| **FSIQ** |  | 0.64 | 0.96 |

RCF: Rey-Osterrieth complex figure; PA: verbal paired associates; STM: Short term memory; (r)PSI: (recovery from) Proactive semantic interference; RSI: Retroactive semantic interference; DIG-F = digit span recall forward; DIG-B: digit span recall backward; VCI: verbal comprehension index; PRI: perceptual reasoning index; WMI: working memory index; PSI: processing speed index; FSIQ: full scale IQ.
