# Supplementary material for: No evidence of reduced capacity during highly demanding cognitive tasks in healthy older adults at electroencephalographic risk of cognitive impairment
Source: PLoS One. 2025 Apr 30;20(4):e0320722. doi: 10.1371/journal.pone.0320722 (PMC12043134; doi:10.1371/journal.pone.0320722)
Supplement: Attachment — Submitted filename: PLOS PONE-D-24-42123 Peer Review_Eder.pdf [file pone.0320722.s006.pdf]

Peer reviewed by Jacob A. Eder., PhD,

For manuscript submission and further recommendations for conciseness of the scope of study.

PLOS ONE

No evidence of reduced capacity during highly demanding cognitive tasks in healthy older adults at electroencephalographic risk of cognitive impairment

--Manuscript Draft—

PONE-D-24-42123

Article Type: Research Article

Full Title: No evidence of reduced capacity during highly demanding cognitive tasks in healthy older adults at electroencephalographic risk of cognitive impairment

Short Title: Electroencephalographic risk of cognitive impairment and cognition

Corresponding Author: Thalía Fernández, Ph.D.

Universidad Nacional Autonoma de Mexico

Querétaro, Querétaro MEXICO

The manuscript demonstrates methodological rigor suitable for PLOS ONE publication standards, based on its design, analysis, and scope of inquiry into EEG biomarkers and cognitive assessment outcomes. The study implements a longitudinal and correlational approach, examining EEG-based markers in tandem with high-demand cognitive assessments to assess risk factors associated with cognitive decline in older adults—a population that often falls within the scope of neurodegenerative studies.

The participant inclusion criteria are meticulously outlined, encompassing individuals within the specified age range who meet neuropsychological benchmarks indicative of healthy cognitive function. Through the application of electroencephalography (EEG) with a specific focus on theta power as a predictive biomarker, the methodology aligns with established neurophysiological protocols. Notably, the use of non-invasive EEG as a predictive tool allows for a nuanced exploration of cognitive reserve and decline, serving as a non-intrusive alternative to cerebrospinal biomarkers, which carry significant procedural risks and costs.

From a neuropsychological perspective, the use of high-cognitive-demand tests—namely, the Visual Short-Term Memory Binding Test (VSTMBT) and the Loewenstein-Acevedo Scale for Semantic Interference and Learning (LASSI-II)—demonstrates an attempt to unveil cognitive decline undetectable by conventional neuropsychological assessments. Both assessments are neuropsychometrically validated tools sensitive to early cognitive dysfunction, and their inclusion reflects an understanding of the need for advanced diagnostic tools in clinical neuropsychology.

Statistical analysis is executed with precision through permutation-based testing, principal component analysis (PCA), and regression modelling, providing a robust framework to

address the potential predictive value of theta power. Corrective measures for multiple comparisons, including false discovery rate (FDR) adjustments, reflect a statistical approach that minimizes Type I error risk, thereby strengthening the validity of the findings.

Although this study's results do not show significant cognitive differences between the groups in either the high-demand or conventional assessments, the findings contribute to the body of literature by challenging the sensitivity of EEG-based markers to predict cognitive deterioration in cognitively healthy older adults. This conclusion aligns with the notion of cognitive reserve, suggesting that neurodegeneration may not immediately manifest behaviourally in individuals with compensatory cognitive mechanisms. Further investigation could explore the potential protective role of higher education levels, which might contribute to these individuals' apparent cognitive resilience.

In summary, the study exemplifies adherence to PLOS ONE's publication criteria, utilizing quantifiable, reproducible, and rigorous techniques. The limitations acknowledged by the authors, such as the educational bias and restricted generalizability, are pertinent and recommend avenues for subsequent research to employ broader, demographically diverse sampling to validate these findings. This study thus establishes a foundation for expanding the application of EEG biomarkers in predictive neuropsychological assessments of age-related cognitive decline.

The study, while methodologically sound, would benefit from addressing several key areas to strengthen its contributions to neuropsychological and neurophysiological literature on age-related cognitive decline. Expanding upon these areas in future research would enhance both the study's impact and its applicability within broader clinical and research contexts. The following recommendations are advised:

1. **Broader Biomarker Integration:**

The study currently relies solely on theta power as the electroencephalographic marker, which, while valuable, may not capture the full range of neurodegenerative processes linked to cognitive impairment. Integrating additional EEG frequency bands (e.g., alpha, delta, beta) could provide a more comprehensive profile of neurophysiological function, particularly in preclinical populations. Furthermore, incorporating other biomarkers, such as cerebrospinal fluid (CSF) amyloid-beta and tau levels, or neuroimaging markers like PET scans for amyloid or tau deposition, would enable a multi-modal approach that could cross-validate EEG findings and strengthen predictive accuracy.

2. **Longitudinal Monitoring:**

Although, this study provides a snapshot of cognitive and EEG measures, longitudinal monitoring over extended periods would reveal whether excessive theta power correlates with cognitive decline over time in the same individuals. Such an approach would enhance understanding of the temporal dynamics of theta power and its potential role as an early marker for neurocognitive disorders. Tracking changes in theta activity and cognitive performance over intervals (e.g., every six months to one year) would help delineate the predictive timeline for cognitive impairment in aging populations.

3. **Diverse and Representative Sample:**

This study's sample shows an educational bias toward highly educated participants, potentially contributing to a heightened cognitive reserve that could mitigate detectable neuropsychological impairment. Future studies should aim for a sample that includes a diverse range of educational backgrounds, as well as socio-demographic diversity, to improve the generalizability of findings. It would be particularly informative to examine whether theta power's predictive value varies across individuals with different baseline cognitive reserves.

4. **Use of Additional Cognitive Assessments:**

While the Visual Short-Term Memory Binding Test (VSTMBT) and Loewenstein-Acevedo Scale for Semantic Interference and Learning (LASSI-II) are suitable for assessing high-cognitive demand and are sensitive to subtle deficits, a broader neuropsychological battery would capture a wider array of cognitive functions potentially affected by neurodegenerative processes. Including tests that assess executive functions, attention, and processing speed—such as the Stroop Task, Trail Making Test, and Wisconsin Card Sorting Test—could reveal more nuanced cognitive impairments associated with excessive theta power. Additionally, the use of computerized cognitive assessments could facilitate finer-grained analysis of reaction times and error patterns, yielding a more detailed cognitive profile.

5. **Exploration of Compensatory Mechanisms and Cognitive Reserve:**

The study suggests that high educational attainment may serve as a proxy for cognitive reserve, potentially enabling individuals to maintain cognitive performance despite neurophysiological alterations. Further exploration of compensatory mechanisms through

functional neuroimaging (e.g., fMRI or MEG during task performance) could clarify the neural correlates of cognitive reserve in older adults with excessive theta power. Identifying brain regions or networks that exhibit compensatory activation patterns would deepen the understanding of how cognitive reserve mitigates the effects of neurophysiological decline and could aid in developing targeted interventions.

6. **Enhanced EEG Protocols and Dynamic Testing Conditions:**

Given that EEG was recorded in a resting state, it may not fully capture task-specific neural activity variations that emerge during cognitive engagement. Employing task-based EEG recordings, particularly during high-cognitive-demand tasks like the VSTMBT and LASSI-II, would yield data on event-related potentials (ERPs) and dynamic EEG responses associated with cognitive performance. ERPs, particularly within theta, alpha, and gamma frequencies, could reveal task-specific deficits not apparent in resting-state EEG and provide a more detailed understanding of cognitive processing under demanding conditions.

7. **Data Sharing and Open Access:**

In line with open science practices, making EEG datasets and cognitive performance scores publicly available (where ethical and permissible) would enable independent verification and meta-analytic integration of findings across studies. While the data availability statement mentions restrictions on the LASSI-II data, public accessibility of non-restricted datasets (e.g., EEG data and VSTMBT scores) would facilitate further research and contribute to building a cumulative evidence base for EEG markers in cognitive impairment.

8. **Consideration of Potential Confounding Factors:**

Factors such as cardiovascular health, diet, and sleep quality—known to impact both EEG measures and cognitive function—are not accounted for in the current analysis. Future studies should consider controlling for or assessing these variables, as they could interact with theta power and cognitive performance. Screening for cardiovascular markers, diet quality, or incorporating self-reported sleep assessments could account for potential confounding effects and provide a more refined understanding of theta power's relationship with cognitive health.

9. **Investigating Non-Linear and Interaction Effects:**

The current analysis appears to focus on linear relationships between theta power and cognitive performance. Wherever, cognitive decline may not exhibit a strictly linear association with theta power, particularly in populations with high cognitive reserve. Future research could investigate non-linear models or explore interactions between theta power and other demographic or neuropsychological variables (e.g., age, education level, and baseline cognitive performance) to uncover complex patterns that linear models may miss.

10. **Developing a Predictive Model with Machine Learning:**

Machine learning (ML) techniques, such as random forests or support vector machines, could be employed to develop predictive models using the study's EEG and neuropsychological data. ML approaches can handle multi-dimensional data and potentially identify latent patterns across theta power, cognitive test scores, and demographic variables, which could improve predictive precision. ML algorithms may thus offer a practical solution for clinicians to assess cognitive risk factors using EEG data, integrating it into a tool for early-stage dementia risk assessment.

Collectively, these recommendations would elevate the study's contributions to the field, offering a more comprehensive understanding of the interplay between EEG biomarkers, cognitive reserve, and neuropsychological assessment in older adults. By addressing these suggestions, future research would refine EEG-based cognitive risk assessment tools, aligning with broader clinical and scientific initiatives for the early detection of neurodegenerative disorders.

RECOMMENDATIONS TO CONSIDER BEFORE PUBLICATION FINALISATION

Based on the thorough examination of this study, the manuscript demonstrates a high level of methodological rigor and scientific integrity, making it a strong candidate for publication. Wherever, while the study aligns well with the standards of PLOS ONE, appropriately and professionally incorporating *specific* revisions before submission could enhance the manuscript's robustness and potential impact. Here is a summary of suggestions to consider:

1. **Expanded Biomarker Assessment**: While theta power is valuable, adding a discussion on the limitations of relying on a single EEG band as a marker and suggesting future studies include additional biomarkers would present a more comprehensive analysis. Mentioning plans to integrate multi-modal biomarker approaches, even if not implemented here, would position this work within the broader context of cognitive impairment research.

2. **Longitudinal Perspective Emphasis**: Reinforcing the potential of a longitudinal follow-up in a future study, particularly considering theta power's predictive window, would give readers a sense of the study's next logical steps. While this is currently a cross-sectional study, emphasizing the importance of observing cognitive and theta power changes over time would underscore its contribution to the field and encourage replication studies.

3. **Task-Based EEG Recommendation**: The manuscript would benefit from acknowledging the limitation of using resting-state EEG alone and suggesting task-based EEG or event-related potential (ERP) testing in future studies. Briefly discussing how task-based EEG might reveal distinct neural activity patterns associated with cognitive effort could add depth to the methodological context.

4. **Broader Sample Demographics**: Addressing the educational homogeneity of the sample more directly, with an acknowledgment of its potential impact on cognitive reserve, could enhance transparency. Proposing a more demographically diverse sample in future studies, as a brief note in the limitations, would also demonstrate attentiveness to generalizability issues.

5. **Confounding Variables:** A short discussion on the influence of confounding factors such as diet, cardiovascular health, or sleep quality, while not feasible within the current scope, would provide context for the findings and inform future studies seeking to disentangle EEG changes specific to cognitive impairment from those influenced by lifestyle factors.

If these suggestions are integrated concisely, the manuscript will present a well-rounded perspective that addresses the current findings while inviting future exploration. Overall, the

paper is near ready for submission, but these minor adjustments would strengthen its reception and significance within the field.
