# Supplementary material for: No evidence of reduced capacity during highly demanding cognitive tasks in healthy older adults at electroencephalographic risk of cognitive impairment
Source: PLoS One. 2025 Apr 30;20(4):e0320722. doi: 10.1371/journal.pone.0320722 (PMC12043134; doi:10.1371/journal.pone.0320722)
Supplement: Attachment — Submitted filename: Response to Reviewers 10-12-2024.doc [file pone.0320722.s008.doc]

December 10, 2024

To Dr. Gabriel G. De La Torre

Academic Editor

PLOS ONE

Dear Dr. Gabriel G. De La Torre,

We are thankful for this first set of revisions provided by the referees. We feel our paper has been remarkably improved by the referees’ suggestions. We provide our responses to each reviewer separately:

**General Notes:**

I. Tables in the manuscript and the supporting information were reformatted to adhere to PLOS ONE specifications. Authors’ contributions were delineated.
II. We included the statement “There was no additional external funding received for this study” in the updated Funding Statement.
III. We will make our data publicly available in the Github repository: https://github.com/JorgeSigg/No-evidence-of-reduced-capacity-during-highly-demanding-cognitive-tasks-in-older-adults-at-EEG-risk-
IV. We double checked and corrected the reference list.
V. The reviewers' suggestions were taken into account.

**Responses to Reviewer 1:**

R1.1.- The study addresses an important gap in the literature by examining the relationship between EEG-based biomarkers of cognitive impairment risk and sensitive neuropsychological tests.

A: Thank you for your comment, we agree that there is a need for addressing this gap. We included your comment as a strength of our study (line 305 - 307): Furthermore, our study's strength is that it addresses an important gap in the literature by examining the relationship between EEG-based biomarkers of cognitive impairment risk and sensitive neuropsychological tests.

R1.2.- The use of both traditional and novel, highly demanding cognitive tests provides a comprehensive assessment. The inclusion of multiple criteria for defining risk groups adds robustness to the analysis. However, to my point of view, there are still some limitations need to be further addressed:

R1.2.1- The statistical approaches, including permutation testing, PCA and multiple comparison corrections, are appropriate and robust. However, the sample size is relatively small, which may limit statistical power to detect subtle differences.

A: Thank you for your comment. We took your advice and stated explicitly this as a limitation of the study (lines 372 - 374): Further limitations include a small sample size and a bias toward higher education, which may limit the statistical power for detecting subtle differences and the generalizability of our results, respectively.

R1.2.2- The cross-sectional design prevents conclusions about the predictive value of excessive theta AP for future cognitive decline.

A: We are sorry for not being sufficiently clear about this. Predictive studies with a longitudinal design were done by Prichep et al. (2006) and Gouw et al. (2017), with a followed up of 10 years and 2 years, respectively. In the present study we considered the established predictive value of theta excess, which was demonstrated by the aforementioned authors. Assuming the predictive value of the excess theta, our study compares two groups of healthy older adults with cognition within normal limits in traditional tests: one group with excess theta (risk group) vs. a group with normal EEG (control), to explore if there are cognitive differences in more demanding cognitive tests between both groups. Adjustments were made in the introduction for clarification purposes (94 - 98): Our study breaks new ground in this field by investigating the potential relationship between EEG-based biomarkers of cognitive impairment risk and neuropsychological tests that have demonstrated high sensitivity to the preclinical phases of neurocognitive disorders. Considering the predictive value of excessive theta activity for cognitive decline, the aim of this study was to fill a significant gap in the current literature.

We are aware of the value of a follow-up assessment of this cohort and added a comment (lines 379 - 382): Furthermore, a follow-up retest in a 2-year timeframe of our cohort sample would be convenient to further assess the predictive accuracy of excessive theta AP on cognitive decline and its correlation with performance on high-demand and traditional neuropsychological tests.

R1.2.3.- The lack of significant differences between groups is intriguing and warrants further investigation. The authors' discussion of cognitive reserve as a potential explanation is appropriate.

A: We are grateful for your comment, and we agree that the lack of cognitive differences in more demanding tasks is most intriguing.

R1.2.4.- It would be valuable to consider other factors that might influence the relationship between EEG markers and cognitive performance, such as lifestyle factors or genetic risk.

A: We are in agreement that there are other factors that influence this relationship, and in other articles we have published the impact that physical activity (Sanchez-Lopez et al., 2018) and cognitive reserve (Ferrari-Díaz et al., 2022) have on this relationship. In fact, we are currently gathering blood samples to establish if there is a relationship between this EEG predictor (excess theta) and genetic (apoE-ε4) and inflammatory (IL-6) risk factors. In the manuscript we made more explicit this study’s limitations (366 - 372): One of the limitations of this study is the lack of use of established biomarkers in CSF, PET, or genotyping, which could confirm that the sample subjects indeed have a predisposition toward neurodegenerative diseases that result in memory impairments, which could have strengthened our results and could cross-validate EEG findings. Despite having controlled for some confounding variables that may influence the EEG (cholesterol, glucose, triglyceride, hemoglobin, and thyroid-stimulating hormone), future studies should also consider sleep hours [49], diet [50], and exercise regime [51] because they have been shown to alter the resting-state EEG.

**Responses to Reviewer 2:**

R2.1.- Broader Biomarker Integration: The study currently relies solely on theta power as the electroencephalographic marker, which, while valuable, may not capture the full range of neurodegenerative processes linked to cognitive impairment. Integrating additional EEG frequency bands (e.g., alpha, delta, beta) could provide a more comprehensive profile of neurophysiological function, particularly in preclinical populations.

A: Thank you for your comments. We incorporated a paragraph in the discussion section about other frequency bands and their relationship to cognitive decline (383 - 392): Even though our research is primarily based on excess theta AP as a risk biomarker for cognitive decline, this is not the only reported EEG risk predictor of cognitive impairment. Prichep et al. [7] established a predictive model of future cognitive decline that included a mix of the mean frequency of the total spectrum on C4, the mean frequency in the delta on T3T5 and P3O1, the AP across all frequency bands diffusely on the right hemisphere and more so on F8, and the theta AP on FP2, F4, and F8. Furthermore, higher delta power, lower alpha power, and lower alpha peak frequency have been associated with clinical progression from preclinical to dementia status [52]. In this study, we compared groups for AP in other frequency bands and found no significant differences. We chose the theta AP excess as a predictor because it is the most deviant from the norms [7] and because of its straightforward implementation in the clinical setting.

R2.2.- Longitudinal Monitoring: Although, this study provides a snapshot of cognitive and EEG measures, longitudinal monitoring over extended periods would reveal whether excessive theta power correlates with cognitive decline over time in the same individuals. Such an approach would enhance understanding of the temporal dynamics of theta power and its potential role as an early marker for neurocognitive disorders. Tracking changes in theta activity and cognitive performance over intervals (e.g., every six months to one year) would help delineate the predictive timeline for cognitive impairment in aging populations.

A: Longitudinal studies are indeed very valuable. We are sorry for not being sufficiently clear about this. Predictive studies with a longitudinal design were done by Prichep et al. (2006) and Gouw et al. (2017), with a followed up of 10 years and 2 years, respectively. In the present study we considered the established predictive value of theta excess, which was demonstrated by the aforementioned authors. Assuming the predictive value of the excess theta, our study compares two groups of healthy older adults with cognition within normal limits in traditional tests: one group with excess theta (risk group) vs. a group with normal EEG (control), to explore if there are cognitive differences in more demanding cognitive tests between both groups. Adjustments were made in the introduction for clarification purposes (94 - 98): Our study breaks new ground in this field by investigating the potential relationship between EEG-based biomarkers of cognitive impairment risk and neuropsychological tests that have demonstrated high sensitivity to the preclinical phases of neurocognitive disorders. Considering the predictive value of excessive theta activity for cognitive decline, the aim of this study was to fill a significant gap in the current literature.

We are aware of the value of a follow-up assessment of this cohort and added a comment (lines 379 - 382): Furthermore, a follow-up retest in a 2-year timeframe of our cohort sample would be convenient to further assess the predictive accuracy of excessive theta AP on cognitive decline and its correlation with performance on high-demand and traditional neuropsychological tests.

R2.3.- Diverse and Representative Sample: This study’s sample shows an educational bias toward highly educated participants, potentially contributing to a heightened cognitive reserve that could mitigate detectable neuropsychological impairment. Future studies should aim for a sample that includes a diverse range of educational backgrounds, as well as socio-demographic diversity, to improve the generalizability of findings. It would be particularly informative to examine whether theta power’s predictive value varies across individuals with different baseline cognitive reserves.

A: We further commented on this topic (lines 338 - 343): A question that remains pending future studies is the impact that education level could have on the predictive accuracy of theta AP as a biomarker of the risk of future cognitive decline. Moreover, the impact of other proxies of cognitive reserve, such as verbal intelligence and leisure activities, should be studied, as well as the neurophysiological substrate of the proxies mentioned above, as this could deepen our understanding of how cognitive reserve mitigates the effects of neurophysiological decline and could aid in developing targeted interventions.

R2.4.- Use of Additional Cognitive Assessments: While the Visual Short-Term Memory Binding Test (VSTMBT) and Loewenstein-Acevedo Scale for Semantic Interference and Learning (LASSI-II) are suitable for assessing high-cognitive demand and are sensitive to subtle deficits, a broader neuropsychological battery would capture a wider array of cognitive functions potentially affected by neurodegenerative processes. Including tests that assess executive functions, attention, and processing speed—such as the Stroop Task, Trail Making Test, and Wisconsin Card Sorting Test—could reveal more nuanced cognitive impairments associated with excessive theta power. Additionally, the use of computerized cognitive assessments could facilitate finer-grained analysis of reaction times and error patterns, yielding a more detailed cognitive profile.

A: We agree that it is a limitation not to include the Wisconsin Card Sorting Test in our neuropsychological battery and not to use computerized tests or virtual reality tests; future studies should incorporate the latter assessment tool for it could detect more subtle differences between groups. We added a comment on this (lines 374 - 377): Another limitation is that we did not assess cognitive deficits using all available cognitive tests for neuropsychological testing or computerized cognitive assessment, which could provide more precise measurements (e.g., reaction times) and, thus, may detect subtle impairments.

R2.5.- Exploration of Compensatory Mechanisms and Cognitive Reserve:
The study suggests that high educational attainment may serve as a proxy for cognitive reserve, potentially enabling individuals to maintain cognitive performance despite neurophysiological alterations. Further exploration of compensatory mechanisms through functional neuroimaging (e.g., fMRI or MEG during task performance) could clarify the neural correlates of cognitive reserve in older adults with excessive theta power. Identifying brain regions or networks that exhibit compensatory activation patterns would deepen the understanding of how cognitive reserve mitigates the effects of neurophysiological decline and could aid in developing targeted interventions.

A: We further discussed this idea (lines 340 - 350): Furthermore, not only cognitive compensatory strategies but also neurophysiological compensatory mechanisms may be involved in adequately performing cognitive tests. Resting-state EEG does not capture task-specific neural activity associated with cognitive processing or the compensatory mechanisms needed to carry out it, as other techniques (EEG, ERP, or fMRI) that explore brain functioning while solving a cognitive task are able to do. For example, neurophysiological compensation could rely on bilateral recruitment of brain regions necessary for task completion, as outlined in the HAROLD model [48], or with different brain network dynamics during task resolution. In fact, Sánchez-Moguel et al. [14] demonstrated that older adults with excessive theta AP had different neurophysiological responses during the Stroop task (no N500 effect) than healthy older adults did, even though the behavioral scores of both groups could not be distinguished.

R2.6.- Enhanced EEG Protocols and Dynamic Testing Conditions:
Given that EEG was recorded in a resting state, it may not fully capture task-specific neural activity variations that emerge during cognitive engagement. Employing task-based EEG recordings, particularly during high-cognitive-demand tasks like the VSTMBT and LASSI-II, would yield data on event-related potentials (ERPs) and dynamic EEG responses associated with cognitive performance. ERPs, particularly within theta, alpha, and gamma frequencies, could reveal task-specific deficits not apparent in resting-state EEG and provide a more detailed understanding of cognitive processing under demanding conditions.

A: We agree that ERP or desynchronization studies while solving high demanding cognitive tests would be interesting research. In the introduction we mentioned 2 ERP studies (Stroop and a syntactic processing task mediated by working memory), in which groups differed in its electrophysiological response. Still, it is important to acknowledge the value that resting EEG has as it reflects the functional integrity of the central nervous system. We added a comment on this (lines 350 - 356): Although differences between these groups have been explored via ERPs during a Stroop task [14] and a syntactic processing task mediated by working memory [13], future studies should evaluate the possibility of changes in ERPs in older adults at the preclinical stage of a potential neurocognitive disorder while high-demand cognitive tests are being performed. This could result in the identification of a very early EEG biomarker for cognitive decline, which would be in line with the ERP alterations reported by Pietto et al. [40] in patients with mild cognitive impairment compared with controls while the VSTMBT was being solved.

R2.7.- Data Sharing and Open Access: In line with open science practices, making EEG datasets and cognitive performance scores publicly available (where ethical and permissible) would enable independent verification and meta-analytic integration of findings across studies. While the data availability statement mentions restrictions on the LASSI-II data, public accessibility of non-restricted datasets (e.g., EEG data and VSTMBT scores) would facilitate further research and contribute to building a cumulative evidence base for EEG markers in cognitive impairment.

A: We welcome this trend towards open science practices and will therefore make all cognitive and sociodemographic measures publicly available, except for the LASSI-II, which we will also include in the github repository (https://github.com/JorgeSigg/No-evidence-of-reduced-capacity-during-highly-demanding-cognitive-tasks-in-older-adults-at-EEG-risk-) once the Mexican normative data article (Roa et al., in preparation) has been published.

R2.8.- Consideration of Potential Confounding Factors: Factors such as cardiovascular health, diet, and sleep quality—known to impact both EEG measures and cognitive function—are not accounted for in the current analysis. Future studies should consider controlling for or assessing these variables, as they could interact with theta power and cognitive performance. Screening for cardiovascular markers, diet quality, or incorporating self-reported sleep assessments could account for potential confounding effects and provide a more refined understanding of theta power’s relationship with cognitive health.

A: We expanded on this topic in the discussion (lines 369 - 372): Despite having controlled for some confounding variables that may influence the EEG (cholesterol, glucose, triglyceride, hemoglobin, and thyroid-stimulating hormone), future studies should also consider sleep hours [49], diet [50], and exercise regime [51] because they have been shown to alter the resting-state EEG.

R2.9.- Investigating Non-Linear and Interaction Effects: The current analysis appears to focus on linear relationships between theta power and cognitive performance. Wherever, cognitive decline may not exhibit a strictly linear association with theta power, particularly in populations with high cognitive reserve. Future research could investigate non-linear models or explore interactions between theta power and other demographic or neuropsychological variables (e.g., age, education level, and baseline cognitive performance) to uncover complex patterns that linear models may miss.

A: We added a comment on this (lines 393 - 396): Moreover, cognitive reserve, cognitive performance, and brain biomarkers appear to exhibit a nonlinear relationship [53]. Thus, using multidimensional models would be an interesting avenue for future research; for example, machine learning approaches could identify latent patterns across theta power, cognitive test scores, and demographic variables, which could improve predictive precision.

R2.10.- Developing a Predictive Model with Machine Learning: Machine learning (ML) techniques, such as random forests or support vector machines, could be employed to develop predictive models using the study’s EEG and neuropsychological data. ML approaches can handle multi-dimensional data and potentially identify latent patterns across theta power, cognitive test scores, and demographic variables, which could improve predictive precision. ML algorithms may thus offer a practical solution for clinicians to assess cognitive risk factors using EEG data, integrating it into a tool for early-stage dementia risk assessment.

A: We integrated this answer in the previous comment (lines 397 - 400).

Sincerely,

Dr. Thalía Fernández
